# Identification and Validation of Reference Genes for RT-qPCR Studies of Hypoxia in Squamous Cervical Cancer Patients

**DOI:** 10.1371/journal.pone.0156259

**Published:** 2016-05-31

**Authors:** Christina S. Fjeldbo, Eva-Katrine Aarnes, Eirik Malinen, Gunnar B. Kristensen, Heidi Lyng

**Affiliations:** 1 Department of Radiation Biology, Norwegian Radium Hospital, Oslo University Hospital, Oslo, Norway; 2 Department of Medical Physics, Norwegian Radium Hospital, Oslo University Hospital, Oslo, Norway; 3 Department of Physics, University of Oslo, Oslo, Norway; 4 Department of Gynaecologic Oncology, Norwegian Radium Hospital, Oslo University Hospital, Oslo, Norway; 5 Institute for Cancer Genetics and Informatics, Norwegian Radium Hospital, Oslo University Hospital, Oslo, Norway; 6 Faculty of Medicine, University of Oslo, Oslo, Norway; Nazarbayev University, KAZAKHSTAN

## Abstract

Hypoxia is an adverse factor in cervical cancer, and hypoxia-related gene expression could be a powerful biomarker for identifying the aggressive hypoxic tumors. Reverse transcription quantitative PCR (RT-qPCR) is a valuable method for gene expression studies, but suitable reference genes for data normalization that are independent of hypoxia status and clinical parameters of cervical tumors are lacking. In the present work, we aimed to identify reference genes for RT-qPCR studies of hypoxia in squamous cervical cancer. From 422 candidate reference genes selected from the literature, we used Illumina array-based expression profiles to identify 182 genes not affected by hypoxia in cervical cancer, i.e. genes regulated by hypoxia in eight cervical cancer cell lines or correlating with the hypoxia-associated dynamic contrast-enhanced magnetic resonance imaging parameter A_Brix_ in 42 patients, were excluded. Among the 182 genes, nine candidates (*CHCHD1*, *GNB2L1*, *IPO8*, *LASP1*, *RPL27A*, *RPS12*, *SOD1*, *SRSF9*, *TMBIM6*) that were not associated with tumor volume, stage, lymph node involvement or disease progression in array data of 150 patients, were selected for further testing by RT-qPCR. geNorm and NormFinder analyses of RT-qPCR data of 74 patients identified *CHCHD1*, *SRSF9* and *TMBIM6* as the optimal set of reference genes, with stable expression both overall and across patient subgroups with different hypoxia status (A_Brix_) and clinical parameters. The suitability of the three reference genes were validated in studies of the hypoxia-induced genes *DDIT3*, *ERO1A*, and *STC2*. After normalization, the RT-qPCR data of these genes showed a significant correlation with Illumina expression (P<0.001, n = 74) and A_Brix_ (P<0.05, n = 32), and the *STC2* data were associated with clinical outcome, in accordance with the Illumina data. Thus, *CHCHD1*, *SRSF9* and *TMBIM6* seem to be suitable reference genes for studying hypoxia-related gene expression in squamous cervical cancer samples by RT-qPCR. Moreover, *STC2* is a promising prognostic hypoxia biomarker in cervical cancer.

## Introduction

Tumor hypoxia is a major factor leading to radiotherapy resistance, metastasis and poor prognosis for many malignant diseases including cervical cancers [1–5]. For measuring hypoxia-related gene expression variations in patient samples, reverse transcription quantitative polymerase chain reaction (RT-qPCR) is a valuable method due to its high sensitivity, flexibility, low cost and ease of use [6–8]. In RT-qPCR analysis, it is important to choose optimal reference genes for data normalization to remove non-biological, experimentally induced variation from the data. For accurate and reliable normalization, multiple reference genes are recommended [6,7,9]. Determination of reference genes for clinical studies is particularly challenging, because the stability of the genes has to be verified in the tissues under investigation and across tumor phenotypes and clinical parameters.

Previous work on reference genes in the uterine cervix has focused on different stages of cervical carcinogenesis by evaluating candidate genes across human papillomavirus (HPV) negative and positive lesions [10], and across normal, precancerous and cancerous samples [11–13]. To our knowledge, suitable reference genes for studying hypoxia-associated gene expression in cervical cancer biopsies have not been reported. Many candidates have been suggested for this purpose from experimental studies where cell lines are cultured under low oxygen concentrations [9,14–17], but only one study on head and neck cancer have utilized the hypoxia status of clinical samples in the validation of such candidates [18]. Tumor-site specific evaluation is required [9,15] and should include associations to clinical features and outcome in addition to hypoxia status.

Most studies searching for reference genes start with a limited panel of about 8–25 candidates selected on the basis of genes commonly used in the literature, which are not necessarily the most stably expressed genes. Utilizing whole genome expression data for an initial evaluation of candidates may be useful for identifying the most promising panel of genes to test in an RT-qPCR assay [19–24]. In the present study, this approach was used to identify suitable reference genes for hypoxia studies in cervical cancer biopsies. A thorough review of the literature identified 422 potential reference genes, of which 410 candidates were present in our data sets and were subjected to an initial evaluation, using gene expression profiles of 150 cervical cancer patients and eight cervical cancer cell lines. Nine genes, not associated with hypoxia or clinical parameters, were further evaluated with RT-qPCR, and three of them were selected as the optimal set of reference genes. The suitability of the reference genes for data normalization was confirmed in studies of three known hypoxia-induced genes in relation to tumor hypoxia status and clinical outcome.

## Materials and Methods

### Ethics statement

The study was approved by The Regional Committee for Medical and Health Research Ethics in South East of Norway (REC S-01129), and written informed consent was achieved from all patients.

### Patient cohorts and tumor specimens

**Fig 1:**
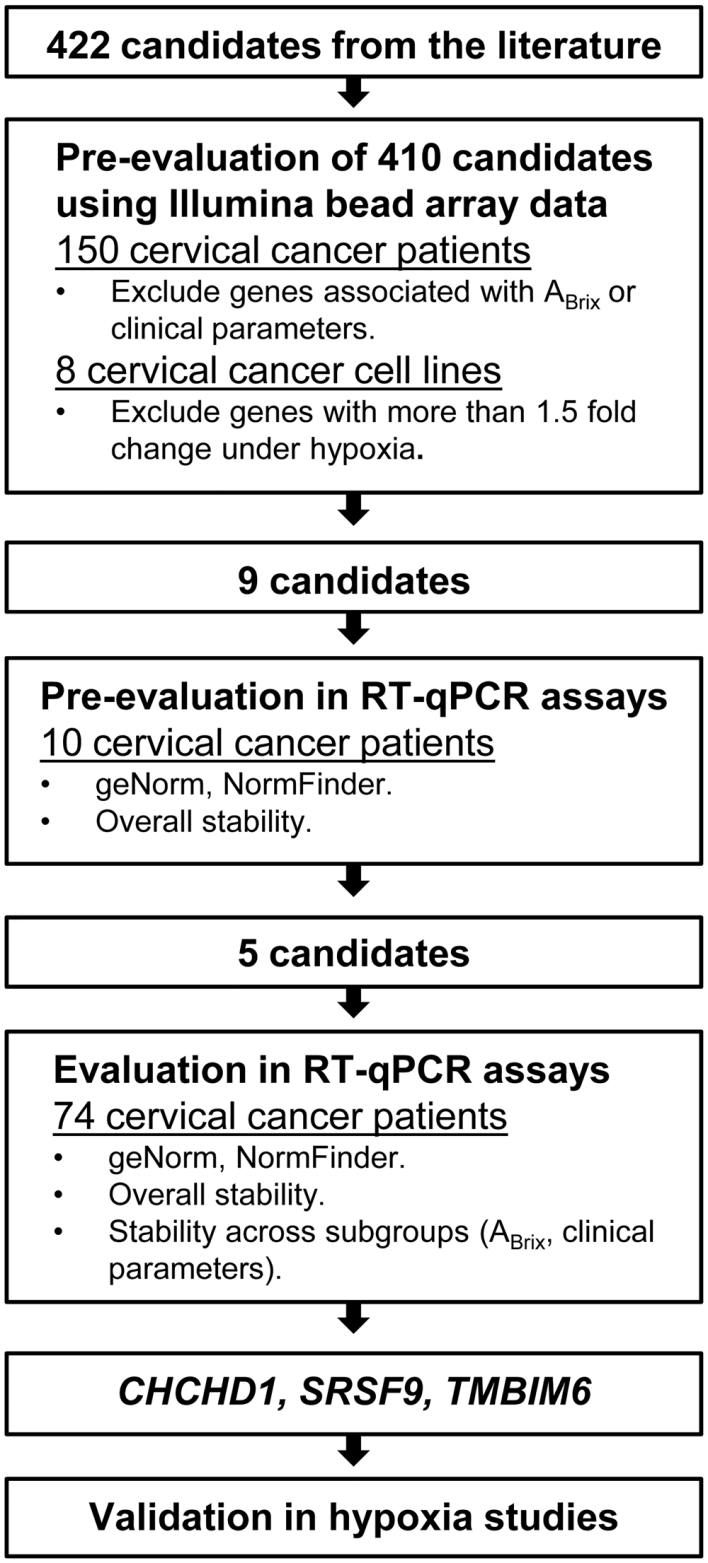
Overview of the study.

Totally 160 patients with locally advanced squamous cell carcinomas of the uterine cervix, prospectively recruited to our chemoradiotherapy protocol at the Norwegian Radium Hospital from 2001 to 2012, were included (Table 1). Illumina gene expression profiles existed for 150 patients (Illumina cohort, Table 1) and were used to select candidate reference genes for testing with RT-qPCR (Fig 1). For 42 of these patients, the hypoxia-associated dynamic contrast enhanced (DCE)-magnetic resonance (MR) imaging parameter A_Brix_ [25] was available and used as measure of tumor hypoxia status. Ten independent patients (RT-qPCR cohort 1) were used for pre-evaluation of nine candidate genes. Moreover, 74 of the 150 patients in the Illumina cohort (RT-qPCR cohort 2) were used for further evaluation and validation of candidates. In addition, 22 biopsies from eight independent patients (2–4 biopsies from each patient) were used to evaluate intra-tumor heterogeneity (heterogeneity cohort).

**Table 1:** Patient and tumor characteristics.

	Illumina cohort (n = 150)		RT-qPCR cohort 1 (n = 10)^a^		RT-qPCR cohort 2 (n = 74)^b^	
Characteristics	No.	%	No.	%	No.	%
**Age (years)**						
Median	54.9		50.2		54.5	
Range	23.8–84.2		25.7–64.2		25.3–80.9	
**FIGO stage**						
1B	10	7	1	10	4	5
2	93	62	5	50	45	61
3	39	26	2	20	22	30
4A	8	5	2	20	3	4
**Tumor volume (cm**^**3**^**)**^c^^,^^d^						
Median	43.8		52.9		44.6	
Range	1.9–321.0		3.5–266.8		2.8–321.0	
**Pelvic lymph node status**^c^^,^^e^						
Positive	64	43	5	50	32	43
Negative	86	57	5	50	42	57
**A**_**Brix**_^f^						
N	42		1		32	
Median	1.51		1.76		1.56	
Range	0.59–3.20		-		0.59–2.33	
**Observation time (months)**^g^						
Median	60		42.9		60	
Range	3.7–60		26.6–60		26.4–60	
**Relapse**						
N	46	31	3	30	32	43
**Non-disease-specific death**^h^						
N	11	7	0	-	5	7

FIGO, Federation International de Gynecologie et d'Obstetrique.

^a^Patients not in Illumina cohort.

^b^Subgroup of patients in Illumina cohort.

^c^Determined from pre-treatment MR images. Tumor volume undetermined for 13 tumors; nine in Illumina cohort, one in RT-qPCR cohort 1, and three in RT-qPCR cohort 2.

^d^Calculated based on 3 orthogonal diameters (a,b,c) as (π/6)abc.

^e^Detected at diagnosis by MRI or CT (n = 13), according to the response evaluation criteria in solid tumors version 1.1.

^f^Determined by pharmacokinetic analysis of pretreatment DCE-MR images based on the Brix model [25].

^g^Based on patients without relapse. Patients dead of other causes are included. Follow up data up to 60 months.

^h^Non-cancer related death before relapse is reported up to 60 months of follow-up.

Tumor volume and presence of pathologic lymph nodes were assessed from pre-treatment MR images. One to four tumor biopsies (approximately 5×5×5 mm) were taken at diagnosis, immediately snap frozen, and stored at −80°C. All patients received external radiation of 50 Gy to the primary tumor and elective areas, 45 Gy to the remaining pelvis, and additional 14 Gy to pathologic lymph nodes. This was followed by brachytherapy of 25 Gy. Concurrent cisplatin (40 mg/m^2^) was given weekly in maximum 6 courses according to tolerance. The patients were followed up according to standard procedure, as described [25].

### Cell culture

Eight human cervical cancer cell lines from the American Type Culture Collection were used to assess hypoxia-responsive genes; HeLa, SW756, C-33 A, C-4I, ME-180, HT-3, SiHa, and CaSki. The cell lines were authenticated by STR/DNA profiling using PowerPlex 21 (Promega, Madison, WI) by Eurofins Genomics (Ebersberg, Germany). The cells were cultured at 37°C in a humidified 5% CO_2_ incubator in Dulbecco’s modified Eagle’s medium (DMEM) with GlutaMAX (Gibco, Life Technologies, Carlsbad, CA) supplemented with 100 U/mL penicillin/streptomycin (Sigma, St. Louis, MO) and 10% fetal bovine serum (FBS, Gibco). Cells were routinely tested for *Mycoplasma* infection. Cells were grown for 24 hours in 10 cm plastic dishes (1–1.8 x 10^6^ cells to obtain ~4 x 10^6^ cells after 48 hours of incubation) before exposure to hypoxic (0.2% O_2_, 5% CO_2_) or normoxic (95% air, 5% CO_2_) conditions for 24 hours at 37°C. An Invivo2200 chamber (Ruskinn Technology Ltd., Bridgend, UK) was used for the hypoxia treatment.

### Gene expression analysis

#### RNA isolation

Total RNA was isolated from cell lines using RNeasy MiniKit (Qiagen, Germantown, MD) and from frozen samples using Trizol reagent (Life Technologies) (Illumina cohort, RT-qPCR cohort 2) or miRNeasy MiniKit (Qiagen) (RT-qPCR cohort 1, heterogeneity cohort). All clinical specimens had more than 50% tumor cells in hematoxylin and eosin-stained sections, derived from the central part of the biopsy. Apart from the heterogeneity cohort, RNA from different biopsies of the same tumor was pooled and RNA concentration was measured using a NanoDrop 2000 spectrophotometer (Thermo Scientific, Waltham, MA). RNA integrity was confirmed by Bioanalyzer (Agilent Technologies, Palo Alto, CA). All samples had an RNA integrity number (RIN) in the range 3.6–9.0 with a median RIN of 7.1.

#### Gene expression by Illumina bead arrays

For whole genome gene expression profiling, Illumina 48K bead arrays with approximately 48000 transcripts were used; HumanWG-6 v3 (patients, cell lines) and HumanHT-12 v4 (cell lines) (Illumina Inc., San Diego, CA). The patient data are part of the data sets used in previous work [25]. cRNA was synthesized, labeled, and hybridized to the arrays. Signal extraction and quantile normalization were performed using the software provided by the manufacturer (Illumina Inc.), and log2-transformed data were used in the analyses. The data were deposited in the gene expression omnibus (GEO) database (GSE75034).

#### Gene expression by RT-qPCR

For RT-qPCR analyses, the 7900HT Fast Real-Time PCR System (Applied Biosystems, Foster City, CA) was applied, and the amplification curves were analyzed using the SDS Software v2.3 (Applied Biosystems). The DNA-free^™^ Kit (#AM1906, Ambion, Austin, TX) was used to remove genomic DNA, according to the manufacturer’s description. Reverse transcription was performed by the High-Capacity cDNA Reverse Transcription kit (Applied Biosystems), which includes random primers, using 2000 ng DNase-treated RNA in a 20 μl reaction. 1 μl cDNA was amplified using the TaqMan Fast Universal PCR Master Mix (2x) and Custom TaqMan Array 96-Well Fast Plates with dried-down TaqMan Gene Expression Assays (Applied Biosystems), or single-tube TaqMan assays and 96-well Fast plates. Thermocycling parameters were as described in the Custom Array protocol, or for single-tube assays: 20 s at 95°C (enzyme activation), 40 thermal cycles of 1 s at 95°C (denaturation) and 20 s at 60°C (annealing and extension). TaqMan assays for candidate reference genes and three hypoxia-induced genes assessed in this study are provided in Table 2. Only pre-developed inventoried TaqMan assays were selected. For evaluating the TaqMan assays, the sizes of the PCR products were determined by gel electrophoresis, using a D1000 ScreenTape^®^ Station (Agilent Technologies). For the three selected reference genes (see below) and the three hypoxia-induced genes, the PCR efficiencies were determined from cDNA dilution curves and showed linear reaction efficiencies (S1 Fig).

**Table 2:** Nine candidate reference genes and three hypoxia-induced genes evaluated in this study.

Gene type	Gene symbol^a^	Entrez gene ID	Accession number	Gene name	Function of encoded protein^b^	TaqMan assay	Exon	Assay location^f^	Amplicon length (bp)
***Candidate reference genes***	*CHCHD1*	118487	NM_203298.2	Coiled-coil-helix-coiled coil-helix domain containing 1	Component of the mitochondrial ribosome small subunit (28S)	Hs00415053_g1^c^	1–2	153	98
	*GNB2L1*	10399	NM_006098.4	Guanine nucleotide binding protein (G protein), beta polypeptide 2-like 1	Possibly involved in protein kinase C (PKC) signaling	Hs00914568_g1^c^	4–5	625	75
	*IPO8*	10526	NM_006390.3	Importin 8	Involved in nuclear import of proteins	Hs00183533_m1^c^	20–21	2615	71
	*LASP1*	3927	NM_006148.3	LIM and SH3 protein 1	Actin-binding protein	Hs00196221_m1^c^	6–7	946	82
	*RPL27A*	6157	NM_000990.4	Ribosomal protein L27a	Component of the 60S subunit of the ribosomes	Hs00741143_s1^d^	5–5	4471	94
	*RPS12*	6206	NM_001016.3	Ribosomal protein S12	Component of the 40S subunit of the ribosomes	Hs00831630_g1^e^	6–6	437	109
	*SOD1*	6647	NM_000454.4	Superoxide dismutase 1, soluble	Binds copper and zinc ions and destroys free superoxide radicals	Hs00916176_m1^c^	2–3	320	138
	*SRSF9*	8683	NM_003769.2	Serine/arginine-rich splicing factor 9	Involved in pre-mRNA splicing	Hs01596548_g1^c^	3–4	674	117
	*TMBIM6*	7009	NM_003217.2	Transmembrane BAX inhibitor motif containing 6	Possible anti-apoptotic activity	Hs00162661_m1^c^	3–4	294	87
***Hypoxia genes***	*DDIT3*	1649	NM_004083.5	DNA-damage-inducible transcript 3	Transcription factor	Hs01090850_m1^c^	1–2	104	78
	*ERO1A*	30001	NM_014584.1	Endoplasmic reticulum oxidoreductase alpha	Involved in disulfide bond formation in the endoplasmic reticulum	Hs00205880_m1^c^	3–4	545	63
	*STC2*	8614	NM_003714.2	Stanniocalcin 2	Secreted glycoprotein that may have autocrine or paracrine functions	Hs01063215_m1^c^	3–4	1812	93

^a^HUGO gene symbols. Previous symbols for *ERO1A* and *SRSF9* were *ERO1L* and *SFRS9*, respectively.

^b^Based on the SOURCE database and published literature.

^c^Probe spans exons.

^d^Both primers and probe map within a single exon.

^e^Amplicon spans exons, probe does not span exons.

^f^Base position contained within the probe.

All samples were run in duplicate, and the mean quantification cycle (C_q_) for each sample was used in the analyses. Samples with mean C_q_ > 37 or standard deviation > 0.4 were excluded. Minus reverse transcriptase controls were tested for residual genomic DNA for all TaqMan assays, and contamination was evaluated by non-template controls without RNA into the cDNA synthesis. All negative controls had undetermined C_q_. The expression level of a target gene was analyzed using the comparative C_q_-method [26], normalizing the C_q_-values of the target gene to a normalization factor calculated as the average C_q_-values of the reference genes; ΔC_q,gene_ = C_q,gene_−(C_q,*CHCHD1*_ + C_q,*SRSF9*_ + C_q,*TMBIM6*_)/3 for the reference genes identified in this study (see below).

### Statistics

The geNorm [6] and NormFinder [7] algorithms were used to evaluate the stability of the candidate reference genes. The geNorm algorithm is based on the principle that the expression ratio of two ideal reference genes is identical in all samples. For each candidate, a stability measure M is calculated as the average pairwise variation of the gene with all other candidates; i.e. the average standard deviation of log2-transformed expression ratios between the genes. The genes with lowest M-values are considered to be most stable. The genes are ranked by stepwise exclusion of the least stable candidate and recalculation of M-values for the remaining genes is performed until the two most stable genes are left. To determine the number of reference genes to include for reliable normalization, pairwise variation between two sequential normalization factors (V_n/n+1_) was calculated for all samples, and the cut-off value for inclusion of an additional gene was set to 0.15 [6]. NormFinder is an ANOVA model-based approach, and was used for estimation of the overall variation of each candidate gene and its variation across patient subgroups by taking into account both the intra- and inter-group variations. The estimated variations are combined into a stability value for each gene, where a low value indicates more stable expression [7].

Spearman’s rank correlation was used for estimating associations between continuous variables, Mann-Whitney U test was used to compare differences in gene expression levels between groups, and COX proportional hazard (PH) univariate analysis was used to assess the relationship of candidate reference genes to clinical outcome. The clinical endpoint was progression free survival (PFS) for follow-up until 5 years. PFS was defined as the time from diagnosis to disease-related death or first event of relapse. Deaths within 2 years after inclusion were regarded as disease-related unless another cause was documented. Patients were censored at their last appointment or at 5 years, and status was assessed at October 3rd 2014. A competing risk model was used for calculating cumulative incidence of disease progression, with death from other causes than cervical cancer as competing event. Eleven out of the 160 patients included died of other causes without experiencing recurrence during follow-up. Since around 1/3 of the patients experienced recurrence, cumulative incidence curves were compared between 1/3 of the cohort with the highest gene expression and 2/3 with the lowest expression. Gray’s test was used to assess differences between the curves. Significance level was 5% unless otherwise indicated, and all P-values were two-sided.

All analyses were performed using R [27] version 3.1.1. For geNorm analyses, the read.qPCR() and selectHKs() functions in the ReadqPCR and NormqPCR packages [28] were applied. For the NormFinder analyses the Normfinder() function provided by Molecular Diagnostic Laboratory, Department of Molecular Medicine, Aarhus University Hospital Skejby, Denmark was used, and the competing risk analyses were performed with the cuminc() function in the cmprsk package [29].

## Results and Discussion

### Selection of candidate reference genes from array-based expression profiles

To identify stably expressed genes in cervical cancer samples, the selection of candidates was limited to genes that have been previously reported as reference genes in the literature [10,11,13,18,30–35], as novel reference candidates based on meta-analysis of large-scale microarray or RNA sequencing data sets [19–23], or present on the TaqMan Express Human Endogenous Control Plate (Applied Biosystems). This resulted in a list of 422 different genes, for which 410 were present on the Illumina array used in the present study (Fig 1, S1 Table), represented by 631 different probes.

The most likely candidates among the 410 genes were identified by utilizing Illumina array-based expression profiles of 150 cervical cancer patients and eight cervical cancer cell lines grown under normoxia and hypoxia. First, candidates were excluded based on their association with a hypoxia response in cervical cancer or with clinical parameters in the patient cohort. Genes for which expression of at least one probe in the clinical data set correlated with the hypoxia-associated DCE-MRI parameter A_Brix_ (n = 42) were removed, as well as genes that were more than 1.5 fold up- or downregulated under hypoxia in at least one of the cell lines (S1 Table). In addition, candidates showing an association between their expression levels and tumor volume, stage, lymph node status, or clinical outcome in 150 patients (S1 Table) were excluded. The removed genes included *GAPDH*, *HMBS*, *EEF1A1*, *ACTB*, *PGK1*, *RPLP0*, and *TBP*, which have been identified as appropriate reference genes in previous studies of cervical carcinogenesis [10–13,36,37], and *B2M*, *HPRT1*, *RPL11*, *RPL37A*, *ACTR3*, and *NDFIP1*, which have been found to be suitable for hypoxia studies in head and neck cancer [18,30]. Totally, 99 different genes represented by 117 probes remained as reference candidates at this stage. To ensure detection of the reference genes in RT-qPCR assays, a further exclusion of genes based on a very low expression level (mean log2 expression of 150 patients < 7) was performed, reducing this number to 89 candidates represented by 101 probes.

In addition to stable expression, the reference gene should ideally have transcript abundance similar to the genes under investigation to increase the sensitivity to detect small gene expression changes [24]. We found that candidates with the lowest coefficient of variation (CV) were generally expressed at high levels, an observation also seen by others [24], whereas most hypoxia-regulated genes from our previous work [25] had a relatively low expression level (data not shown). A final panel of genes to be evaluated by RT-qPCR was therefore selected to represent different expression levels in addition to keeping the CV as low as possible. Candidates were also chosen in such a way that they represented different pathways or functional classes in order to minimize the risk of selecting co-regulated genes [6]. Finally, the availability of a suitable pre-designed and validated TaqMan-assay was used as a selection criterion. The panel of nine candidates to be tested in RT-qPCR assays is listed in Table 1, and had CVs in the range of 0.01–0.09 and mean log2 expression levels in the range of 7.4–14.0 in the clinical data set. Among these genes, *GNB2L1* has been previously used as reference gene in hypoxia studies of head and neck cancer [18].

### Pre-evaluation of 9 candidate reference genes by RT-qPCR

**Fig 2:**
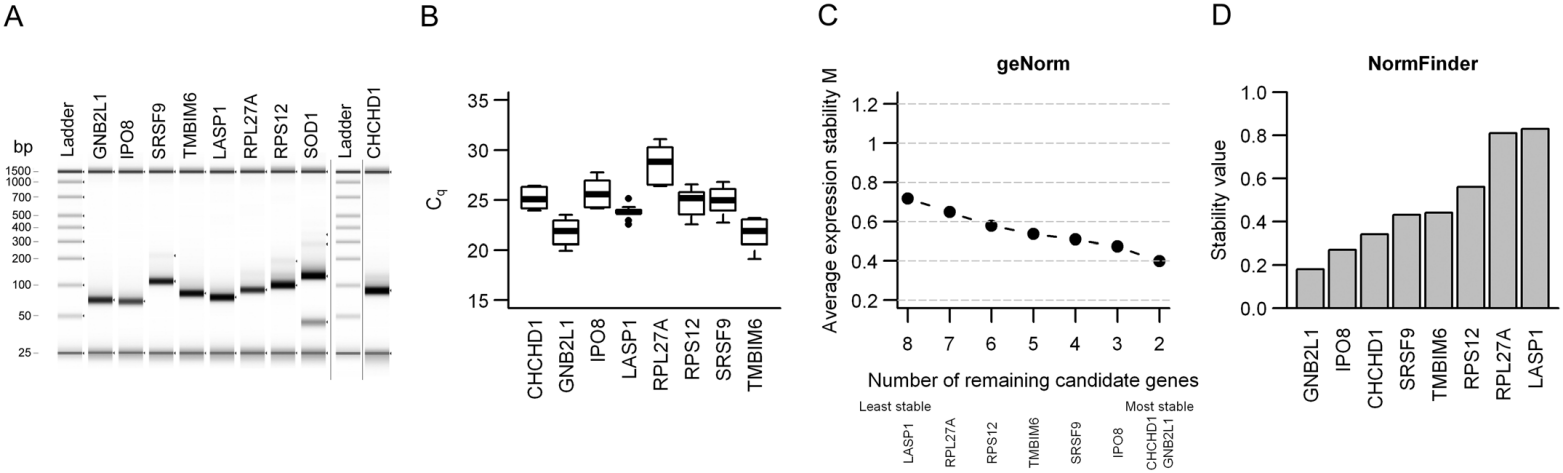
Pre-evaluation of 9 candidate reference genes by RT-qPCR in 10 patients. (**A**) Gel electrophoresis of the PCR products for nine candidate reference genes in one patient. Lower (25 bp) and upper (1500 bp) markers are shown in each lane. Gene symbols are indicated. The figure is a composite image where *CHCHD1* is from a separate image and the ladder from each image is shown. Vertical lines indicate cropping of the image or different images. (**B**) Box plots of the arithmetic means of duplicate C_q_-values for eight candidate reference genes in 10 patients. Boxes indicate the interquartile range (IQR) with median as the black center bar. Extended vertical bars represents 1.5 x IQR below the first quartile and 1.5 x IQR above the third quartile, and circles mark suspected outliers. (**C**) geNorm analysis of eight candidate reference genes. Average expression stability (M) of the remaining candidates after stepwise removal of the least stable gene is shown. The least stable gene in each step is indicated below. (**D**) Stability value of each of the eight candidate reference genes from the NormFinder analysis, where a low value indicates more stable expression.

To ensure that the candidates could be properly detected in a TaqMan assay and possibly further limit the panel, a pre-evaluation of the nine genes was performed in 10 patients (RT-qPCR cohort 1, Table 1, Fig 1). The TaqMan assays were first evaluated by gel electrophoresis of the PCR-products from one patient (Fig 2A). For all assays, a strong band of expected size was seen. However, for *SOD1* an additional weaker band of smaller size that might represent primer dimers also appeared, indicating that the assay was not working optimally. *SOD1* was therefore excluded in the further analyses.

The eight remaining candidates were detected with C_q_-values ranging from 19.1 to 31.1, where *GNB2L1* and *TMBIM6* were the most abundant and *RPL27A* the least abundant transcript (Fig 2B). geNorm and NormFinder analyses were performed to rank the genes according to the overall stability across the ten patients (Fig 2C and 2D). The average expression stability M from the geNorm analysis ranged from 0.72 using all eight genes to 0.4 for the two most stable candidates (Fig 2C). The ranking of the genes in the NormFinder analysis was almost identical to the geNorm ranking (Fig 2D). For a more thorough stability analysis, including assessment of the stability across patient subgroups, and determination of the optimal number of reference genes to include for normalization, the five most stably expressed genes as determined by the two methods, i.e. *CHCHD1*, *GNB2L1*, *IPO8*, *SRSF9* and *TMBIM6*, were examined further.

### Determination of an RT-qPCR normalization factor

**Fig 3:**
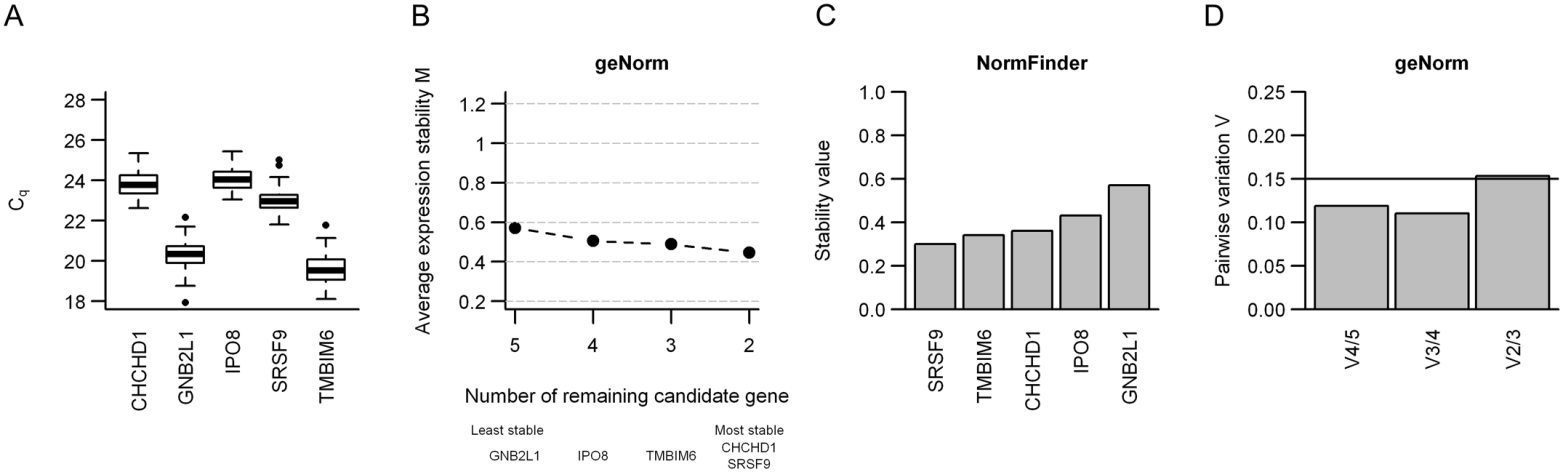
Evaluation of overall stability of 5 candidate reference genes by RT-qPCR in 74 patients. (**A**) Box plots of the arithmetic means of duplicate C_q_-values for five candidate reference genes in 74 patients. Boxes indicate the interquartile range (IQR) with median as the black center bar. Extended vertical bars represent 1.5 x IQR below the first quartile and 1.5 x IQR above the third quartile, and circles mark suspected outliers. (**B**) geNorm analysis of five candidate reference genes. Average expression stability (M) of the remaining candidates after stepwise removal of the least stable gene is shown. The least stable gene in each step is indicated below. (**C**) Stability value of each of the five candidate reference genes from the NormFinder analysis, where a low value indicates more stable expression. (**D**) geNorm pairwise variation (V) analysis to determine the sufficient number of reference genes in the normalization factor. Pairwise variation for two sequential normalization factors (V_n/n+1_) from the two most stable genes to all five genes. The horizontal line indicates the cut-off value (V = 0.15), for which inclusion of more genes has no significant effect on the normalization factor.

The five remaining candidates were evaluated by RT-qPCR analysis in 74 patients (RT-qPCR cohort 2, Table 1, Fig 1), resulting in C_q_-values between 17.9 and 25.4 (Fig 3A). geNorm and NormFinder analyses showed similar ranking of the genes according to the stability across the patients (Fig 3B and 3C). From the geNorm analysis, the average expression stability M ranged from 0.57 using all five genes to 0.45 using the two most stable candidates.

To evaluate the sufficient number of genes required for reliable normalization, a stepwise procedure was utilized by including genes sequentially into the normalization factor according to their increasing M-values until no significant contribution was achieved. The pairwise variation between the normalization factors with three and four genes were below the cut-off value (V_3/4_ < 0.15), indicating that the three candidates with lowest M-value, *CHCHD1*, *SRSF9* and *TMBIM6*, are sufficient for normalization (Fig 3D). These candidates were also found to be the three most stable genes in the NormFinder analysis (Fig 3C). Moreover, their average M-value was 0.49 (Fig 3B), and below the range of 0.5–1 that should be required when evaluating reference genes in cancer biopsies [38].

**Fig 4:**
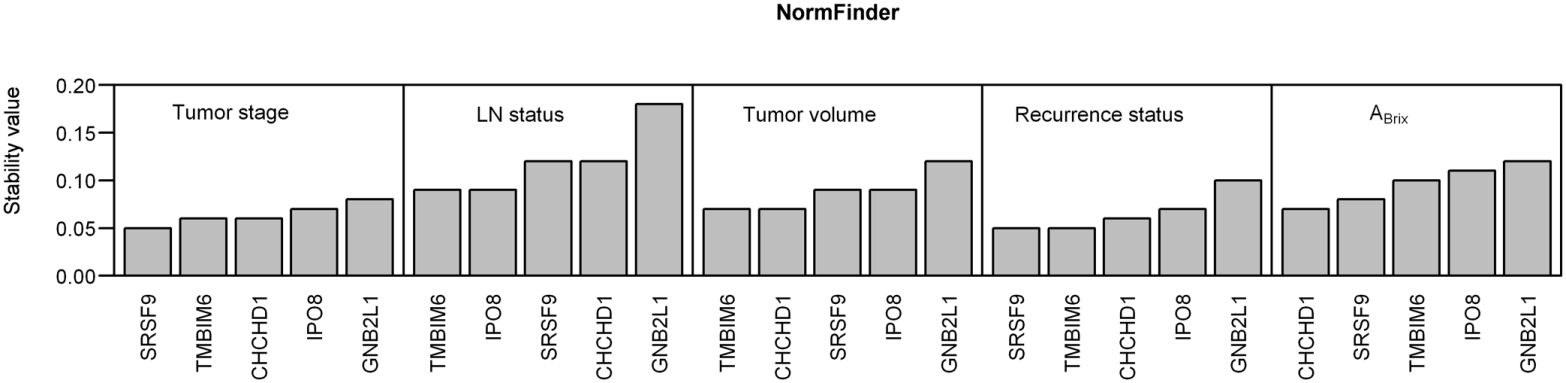
Evaluation of stability across subgroups for 5 candidate reference genes by RT-qPCR in 74 patients. NormFinder analyses of the stability of five candidate reference genes across patient subgroups. The subgroups assessed were: low (n = 49) and high (n = 25) tumor stage (FIGO 1B-2B vs. 3A-4A), with (n = 32) and without (n = 42) lymph node (LN) involvement at diagnosis, below (n = 36) and above (n = 36) a median tumor volume of 44.6 cm^3^, with (n = 32) or without (n = 42) treatment recurrence at five years, and different hypoxia status represented by below (n = 16) and above (n = 16) a median A_Brix_.

We further evaluated the stability of the five candidates across patient subgroups using the NormFinder algorithm (Fig 4). In this analysis, *CHCHD1*, *SRSF9* and *TMBIM6* also appeared as the optimal reference genes. They were the most stable candidates across patients with low and high tumor stage or volume and with and without recurrence, and for patients with and without lymph node involvement at diagnosis the three candidates were among the four most stable genes. Moreover, in analysis of the tumor hypoxia status, *CHCHD1*, *SRSF9* and *TMBIM6* were the most stable candidates across patients with high and low A_Brix_ (Fig 4). To further validate the stability of these three genes, geNorm analysis on RT-qPCR data from normoxic and hypoxic samples from eight cervical cancer cell lines was performed. The average M-value for the three genes were 0.79, indicating stable expression across normoxic and hypoxic culture conditions [38]. Thus, *CHCHD1*, *SRSF9* and *TMBIM6* seemed to be well suited for calculating a normalization factor and were selected for validation in studies of hypoxia-induced gene expression (Fig 1).

### Validation of *CHCHD1*, *SRSF9* and *TMBIM6* as reference genes in hypoxia studies

**Fig 5:**
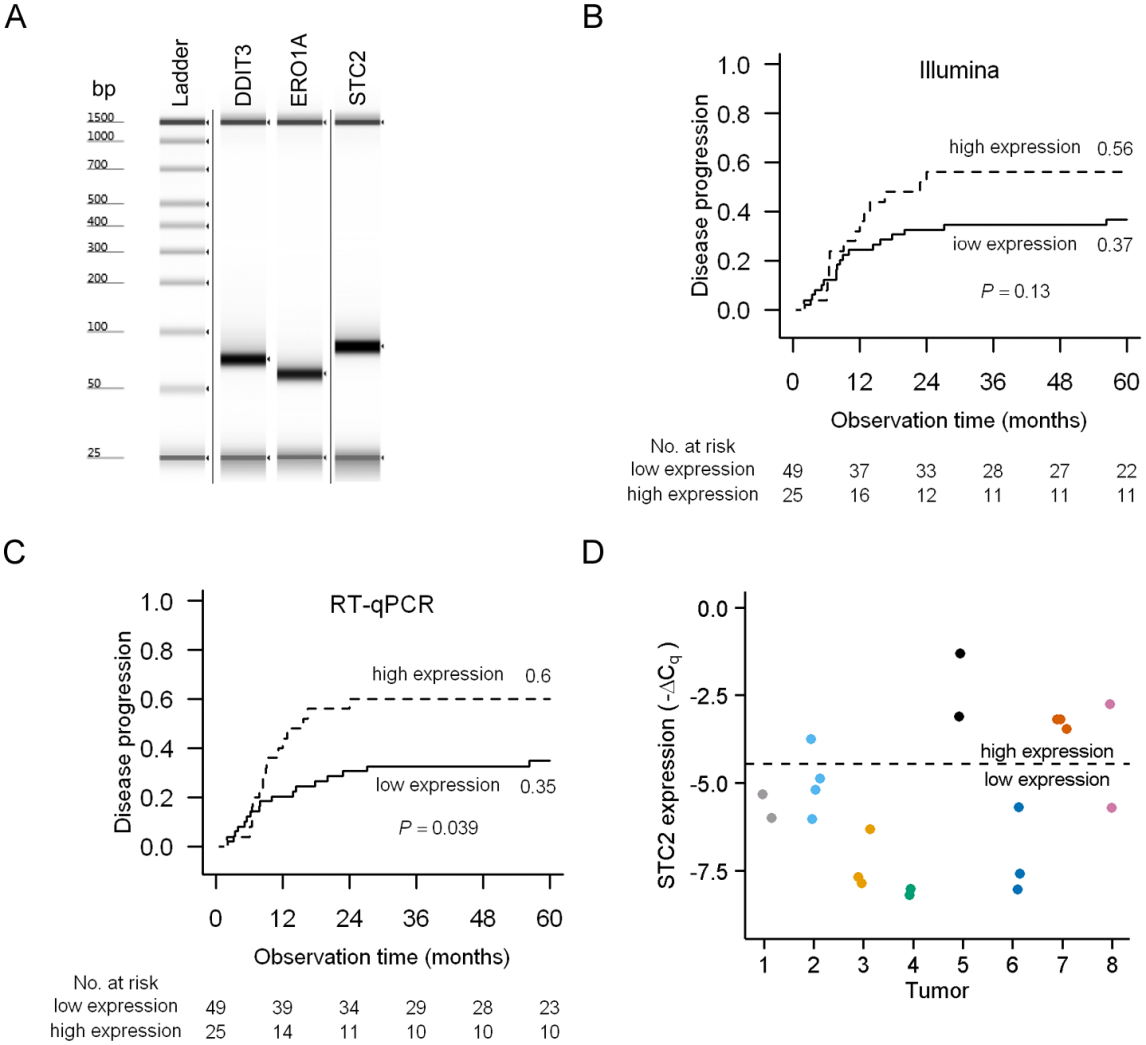
*CHCHD1*, *SRSF9* and *TMBIM6* as reference genes in studies of hypoxia-induced gene expression in cervical cancer patients. (**A**) Gel electrophoresis of the PCR products for the three hypoxia-induced genes *DDIT3*, *ERO1A*, and *STC2*. Lower (25 bp) and upper (1500 bp) markers are shown in each lane. The figure is derived from one image, and vertical lines indicate cropping of the image. Cumulative incidence of disease progression for 74 patients divided into low (< 67% percentile) and high (≥ 67% percentile) *STC2* expression based on (**B**) Illumina expression data and (**C**) RT-qPCR data normalized with *CHCHD1*, *SRSF9* and *TMBIM*6 (-ΔC_q_). 60 months recurrence probability, P-values from Gray’s test and number of patients at risk are indicated. Death from other causes than cervical cancer was included as a competing event (n = 5). (**D**) Intra-tumor variability in *STC2* expression levels measured by RT-qPCR across eight independent tumors with 2–4 biopsies per tumor, i.e. in total 22 biopsies. Measurement of *STC2* was unsuccessful for one of the biopsies for tumor 4. *STC2* data were normalized with *CHCHD1*, *SRSF9* and *TMBIM*6. The samples were classified into a high and low expression group using the same cut-off as in Fig 5C (i.e. –ΔC_q_ = -4.46). Different biopsies from the same tumor have been plotted with the same color to ease the interpretation of the figure.

RT-qPCR analyses were performed for three genes that we have previously reported to be induced by hypoxia in cervical cancer cell lines and associated with A_Brix_ in cervical cancer [25], i.e. *DDIT3*, *ERO1A* and *STC2*. The RT-qPCR cohort 2 of 74 patients, for which we had Illumina expression data, was utilized. Gel electrophoresis of the PCR products showed that the TaqMan assays worked properly, generating a single specific product of the expected size for each of the three genes (Fig 5A). The expression levels of *DDIT3*, *ERO1A* and *STC2* were calculated by normalizing the C_q_-values to the average C_q_-value of *CHCHD1*, *SRSF9* and *TMBIM*6. The -ΔC_q_-value was shown to be highly correlated with the Illumina data for all three genes (P < 0.001; ρ from 0.78 to 0.83). Further, significant negative correlations between -ΔC_q_ and A_Brix_ were found, in agreement with the correlations obtained with Illumina data (Table 3). The identified reference genes therefore seem suitable for measurement of hypoxia-induced changes in cervical cancer.

**Table 3:** Association between gene expression and tumor hypoxia status (A_Brix_).

Gene symbol^a^	Illumina arrays (log2)		RT-qPCR (-ΔC_q_)^b^	
	ρ	P	ρ	P
***DDIT3***	-0.46	0.009	-0.44	0.012
***ERO1A***	-0.43	0.015	-0.38	0.035
***STC2***	-0.41	0.021	-0.43	0.015

Correlation coefficient (ρ) and P-value from Spearman’s rank correlation analysis on 32 cervical cancer patients.

^a^HUGO gene symbols.

^b^Normalized with *CHCHD1*, *SRSF9* and *TMBIM*6.

Since hypoxia is known to be associated with aggressiveness in cervical cancer [3–5], we evaluated the prognostic value of *DDIT3*, *ERO1A* and *STC2* expression in the Illumina and RT-qPCR data sets. Based on the Illumina data of 150 patients, a statistically significant increased risk of disease progression was found for patients with the highest expression of *DDIT3* or *STC2* compared to the others (P = 0.047 and P = 0.015, respectively, Gray’s test; data not shown), whereas *ERO1A* expression was not associated with outcome (data not shown). In the RT-qPCR cohort 2 of 74 patients, the strongest association to outcome was found for *STC2* in both data sets, and for the RT-qPCR data the relationship was statistically significant (Fig 5B and 5C). This further validated the suitability of *CHCHD1*, *SRSF9* and *TMBIM*6 as reference genes in hypoxia studies, and suggested that high *STC2* expression is associated with chemoradiotherapy failure, in accordance with a previous study on cervical cancer [39]. Moreover, by RT-qPCR analysis *STC2* was verified as strongly hypoxia-induced in most of the eight cervical cancer cell lines (S2 Fig), supporting the finding that *STC2* might be an important hypoxia-regulated gene in cervical cancer. *DDIT3* and *ERO1A* were also verified as hypoxia-induced in cell lines, although a smaller induction was in general observed compared to *STC2* (S2 Fig). Further, to address the intra-tumor heterogeneity of *STC2* expression, its expression level in 22 biopsies from eight independent tumors was measured. The stability of the three reference genes was validated in these new samples with an average M-value of 0.89 in geNorm analysis. For most of the tumors all biopsies were classified into either the low- or high *STC2* expression group (Fig 5D), further supporting a potential of this gene as hypoxia biomarker. Associations between *STC2* expression and treatment outcome have also been shown for other cancer types, like gastric cancer [40], nasopharyngeal carcinoma [41], renal cell carcinoma [42], esophageal squamous-cell carcinoma [43], and colorectal cancer [44]. Studies in cell lines have identified *STC2* to be involved in cellular growth, migration, and invasion, both under normoxic [43,45,46], and hypoxic conditions [47–50]. Taken together, *STC2* might be a molecular biomarker of hypoxia-related tumor aggressiveness in cervical cancer that warrants further investigation.

## Conclusions

We have identified and validated *CHCHD1*, *SRSF9* and *TMBIM6* as reference genes for studying hypoxia-related gene expression in fresh-frozen clinical samples from squamous cell carcinoma of the uterine cervix by RT-qPCR. We have also shown that high *STC2* expression was associated with chemoradiotherapy failure in our cohort, implying that *STC2* might be a useful prognostic hypoxia biomarker in cervical cancer.

## Supporting Information

S1 Fig Standard curve and PCR efficiency for three reference genes and three hypoxia-induced genes. (PDF)

S2 Fig Hypoxia-induced gene expression changes for *DDIT3*, *ERO1A* and *STC2* in eight cervical cancer cell lines. (PDF)

S1 Table List of 422 candidate reference genes selected from the literature and their association to hypoxia and clinical parameters. (XLSX)
