# Supplementary material for: Identification and Validation of Reference Genes for RT-qPCR Studies of Hypoxia in Squamous Cervical Cancer Patients
Source: PLoS One. 2016 May 31;11(5):e0156259. doi: 10.1371/journal.pone.0156259 (PMC4887009; doi:10.1371/journal.pone.0156259)
Supplement: S1 Fig — (PDF) [file pone.0156259.s001.pdf]

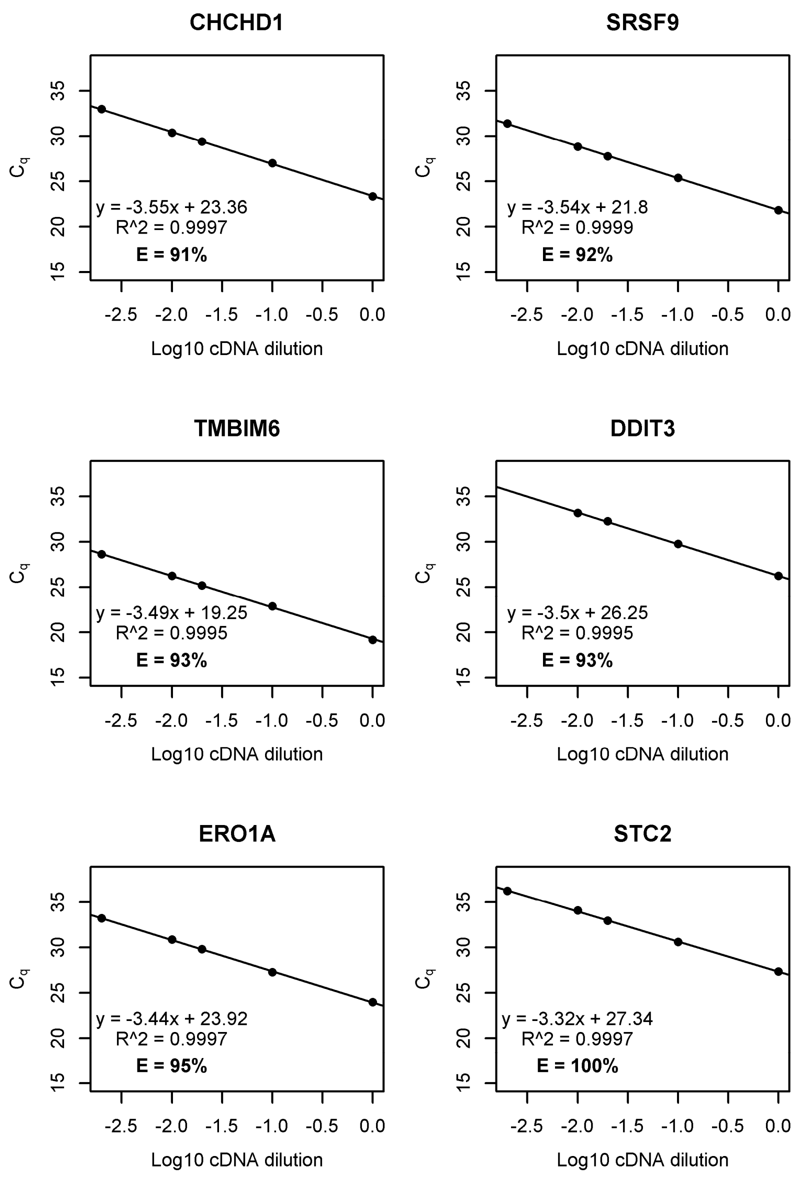

S1 Fig. Standard curve and PCR efficiency for three reference genes and three hypoxia-induced genes. Average C_q values from qPCR duplicates for five cDNA concentrations (0.2 – 100 ng total RNA converted to cDNA into the qPCR reaction) using cDNA from SiHa cells grown under normoxic conditions. For *DDIT3*, the C_q value for the most diluted sample was not determined .The PCR efficiencies were determined from the cDNA dilution curves using the formula $E = [10^{(-1/slope)}-1]$.
