# Supplementary material for: Identification and Validation of Reference Genes for RT-qPCR Studies of Hypoxia in Squamous Cervical Cancer Patients
Source: PLoS One. 2016 May 31;11(5):e0156259. doi: 10.1371/journal.pone.0156259 (PMC4887009; doi:10.1371/journal.pone.0156259)
Supplement: S2 Fig — (PDF) [file pone.0156259.s002.pdf]

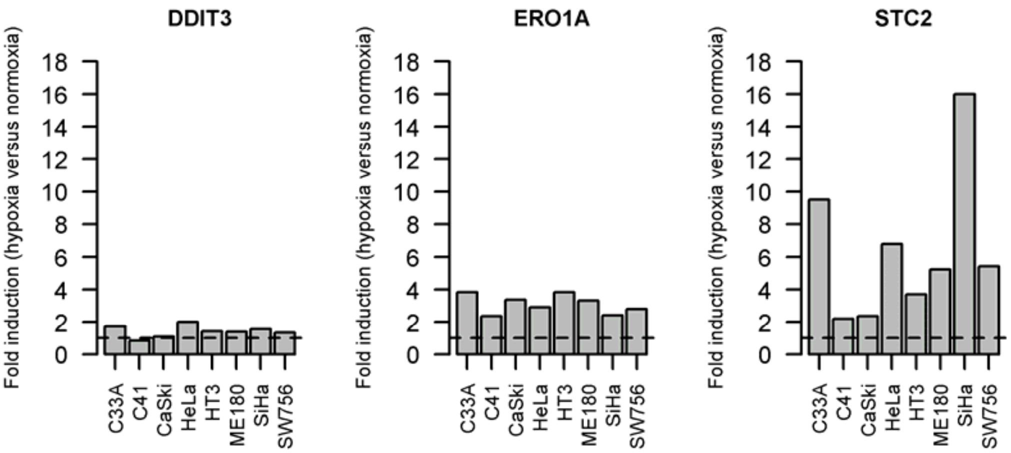

S2 Fig. Hypoxia-induced gene expression changes for *DDIT3*, *ERO1A* and *STC2* in eight cervical cancer cell lines. C_q values for each of the three genes are normalized to the average C_q values of the three reference genes *CHCHD1*, *SRSF9* and *TMBIM6* yielding ΔC_q values. $\Delta\Delta C_q$ values are calculated based on ΔC_q under hypoxic condition minus ΔC_q under normoxic conditions, and fold induction is $2^{-\Delta\Delta Cq}$. The bars represent the average of two technical replicate experiments run in duplicate in the qPCR step. The stippled lines indicate a fold induction of 1 under hypoxic conditions compared to normoxic conditions.
